# The After-Care of Asylum Convalescents

**Published:** 1887-01-22

**Authors:** 


### The After-Care of Asylum Convalescents.

### An Interview with Lord Brabazon.

Not a little interest having been aroused amongst medical men and the philanthropic public by the efforts which have lately been made to start an Association for the care of poor females discharged as convalescents from the various county and other asylums in the country, a representative of The Hospital waited upon Lord Brabazon a few days ago to gather some particulars with reference to the movement. He was fortunate also to meet at the same time Mr. H. Thornhill Roxby, the secretary of the Society.

"This Association is not quite a new one, I believe, Lord Brabazon?"

"Oh, no. It was started in 1879, and the great Lord Shaftesbury, whose position as chairman of the Lunacy Commissioners for over fifty years enabled him to appreciate the need for such a Society, became its president. He expressed a strong opinion that a Home for mental convalescents was a necessity. But up to the present not much has been done, and we are now trying to put more life into the Society. Its title, 'The After-care Association for Poor and Friendless Female Convalescents on Leaving Asylums for the Insane,' is a long one, is it not? But it will be generally known, I expect, as simply the 'After-care Association.' The object of the Society is to establish Convalescents' Homes in connection with our county and borough lunatic asylums, for the purpose of receiving within their walls poor and friendless female convalescents from those asylums. There is a pressing need for this, inasmuch as there is no institution at present established which will take in such convalescents."

"Well, what becomes of these poor people now?"

"Their condition is a very sad one, for you will readily understand why they are refused admission to the ordinary Convalescent Homes. People are afraid of receiving persons who have been inmates of lunatic asylums, from a fear that they might not be thoroughly restored to health. Still more readily will you understand that mental convalescents are not fit to enter on the responsibilities of life immediately on their dismissal from the doors of the lunatic asylums. People will not take them into their houses in any capacity, so much do they dread a return of the malady from which they have been suffering. Then, again, these convalescents have been for a more or less lengthened period within the walls of an asylum, and have often lost the thorough power of knowing how to make their way in the world. They have been well cared for, and waited upon, so that they are sometimes very like children when the period of their incarceration has expired, and urgently require supervision and care."

"I presume it is difficult to trace the future of these people at present."

"Many of them reach the prison or are condemned to a worse fate. On this point the testimony of the Rev. J. W. Horsley, late chaplain of Her Majesty's prison at Clerkenwell, is very valuable. He says, 'I rejoice to hear that some at least of those who are interested in the future care of those who have been mentally afflicted are considering the desirability of establishing some Home into which convalescents may go for awhile after their discharge from the asylum. I have not seldom noticed in prisons cases of men, and especially perhaps of women, who have been discharged from asylums, who have shown themselves to be not fit for the ordinary duties or the inevitable battles of life. Hence, without actual criminality or responsibility, they have found their way to Clerkenwell as "wandering abroad," or for attempting suicide; and in many of these cases I am morally certain that a judicious mingling of freedom and restraint, combined with kindly and watchful care for awhile after their discharge, would have prevented their relapse. They are neither fit for the asylum nor for liberty. Let them be made fit for liberty, and they will not again be fit for the asylum.'"

"That last sentence seems to put the whole matter in a nutshell."

"Yes," interposed Mr. Roxby. "Only within the last few days I have heard of a very sad case. A poor woman was recently discharged from an asylum near London, convalescent, it is true, but quite unfit for full liberty and the duties of life. She will be bound to be back there before very long; and if we only had a Home for cases like this, probably they would never return to the asylum, and would become useful members of society."

"We all know," resumed Lord Brabazon, "the benefit of such Convalescent Homes in connection with the London hospitals, where patients who have recovered from diseases of the body are sent to recruit health and strength before they return to their daily life. How much greater, then, must be the need of Convalescent Homes for those who have been afflicted with mental derangement, and are, by God's mercy, restored to health in mind and brain! Yet no such Home exists; although I believe there is one convalescent institution where occasionally such cases are taken. Try and realise the condition of a patient sent away from a locked cell, and the ever-careful watchfulness of the attendants of the asylum, straight into the unrestrained freedom and the bewildering excitement and bustle of every-day life. Many of the patients come from the poorest rank of life; not a few are homeless, friendless, and helpless. If the charitable public will only think of these matters, I feel sure we shall not plead in vain for funds to enable our Association to commence its work. What *can* the discharged patients do when they leave the asylum? Are they fit to enter at once into domestic or other services, that is, supposing any stranger could be found to receive them? The suddenness of the change oftentimes induces a speedy revisitation of their calamity."

"What is the numerical extent of the class amongst which you desire to conduct your operations?"

"Well, there are about thirty thousand women of various callings and conditions of life in lunatic asylums in England and Wales, but we should only deal with those who are too poor to be properly cared for by their own relatives, or who are absolutely friendless—but these will furnish a sufficiently wide field. I mentioned just now the interest Lord Shaftesbury took in the matter. He was exceedingly anxious to establish a Convalescent Home for these cases, and we are most desirous to carry out the views of so great an authority."

"In what position does your Association stand now? You succeeded the late Earl as president?"

"Yes. I should mention that Her Royal Highness Princess Christian has graciously consented to become the patroness; and we have a strong committee, including Lady Frederick Cavendish, the Dowager Lady Lyttleton, Mrs. Gladstone, Sir Andrew Clark, Dr. Ogle, Dr. Savage, Dr. D. Hack Tuke, Dr. Richardson, and others. Dr. Shaw, of the Banstead Asylum, is the treasurer, and Mr. Gerard Van de Linde, of 12, Lawrence Pountney Lane, is our auditor; the Rev. Henry Shaw, the chaplain of Colney Hatch Asylum, is the hon. secretary, and Mr. Roxby's address is Emblewood, Osbaldeston Road, Stoke Newington."

"You had better add," said Mr. Roxby with a secretarial eye to business, "that our bankers are the Union of London, Regent Street branch. People will often send their contributions to a bank, when they wouldn't send to a private address."

"What means have you taken to give publicity to your requirements, Lord Brabazon?"

"As I said before, we are really only just starting; and the letter which I sent to *The Times* the other day is, as one may say, the first step. We have had drawing-room meetings in this house, at Sir Andrew Clark's, at Dr. Bucknill's, at Dr. Ogle's, at Lord Cottesloe's, and at Bethlehem Hospital, but they were only preliminary meetings. We hope now to get to work in real earnest."

"And what is the amount of your immediate demand upon the purse of the charitable?"

"We are making a strenuous effort to raise the sum of £2,000 for the purpose of maintaining a Convalescent Home for Discharged Inmates, if we can only obtain the offer of a suitable house and grounds *gratis*."

"The latter will probably be more difficult to obtain than the former? Two thousand pounds should be easily raised."

"I don't know. The house question is not so difficult as you suppose. There are more than two hundred Homes on the register of the Charity Organisation Society, but not one appears to be specially designed and available for persons recovering from mental derangement. Our committee hope and believe, however, that if our requirement is made publicly known, it will be possible to find some convalescent home or institution which, from want of adequate support, is more or less in a failing condition, and the owners or managers of which would be glad to let us have the use of it free for the purposes which we have in view."

"Quite so. I suppose the Association will not confine itself to the provision of one or more Homes?"

Oh, no. It will seek to benefit the discharged female lunatics in other ways. As a means of providing them with the needful change of scenery and air—often so beneficial on recovery from ordinary sickness—we hope to be able to board out some of our convalescents in country places. Then, again, we want to raise funds to give grants of money and clothing, of which latter some of the poor women are dreadfully deficient on their discharge. In some of the asylums there is a sort of Royal Bounty Fund, out of which out-going patients are relieved to the extent of a few shillings. But this is so small as to be little better than useless, and, after all, leaves untouched the greatest need of all, for some kindly supervision, help, and counsel to the poor women thus suddenly thrown afresh into the bustling world. Finally, the Association will try and find suitable employment for discharged inmates of lunatic asylums as they become fitted for it. There is no question about the real need of the work that we are going to try and do; and I have no doubt that, as our aims and objects become known, we shall not lack the pecuniary support which is necessary. But we can do nothing without money."

